# Comparison Between the Effect of Topical Platelet-Rich Fibrin and Absorbable Gelatin Sponge on Graft Uptake in Type 1 Tympanoplasty

**DOI:** 10.7759/cureus.106090

**Published:** 2026-03-29

**Authors:** Deepak K Gupta, Diksha Sahu, Neena Chaudhary, Gowtham Palaniyappan, Neeti Khunger

**Affiliations:** 1 Otolaryngology-Head and Neck Surgery, Vardhman Mahavir Medical College and Safdarjung Hospital, New Delhi, IND; 2 Dermatology, Vardhman Mahavir Medical College and Safdarjung Hospital, New Delhi, IND

**Keywords:** chronic otitis media, platelet-rich fibrin, post-aural approach, type 1 tympanoplasty, underlay technique

## Abstract

Background: Chronic otitis media (COM) is an inflammatory process in the middle-ear space that results in long-standing or permanent changes in the tympanic membrane. Platelet-rich fibrin (PRF) is a unique platelet concentrate that is being used in various fields due to its capability to augment healing.

Aims: Our study aimed to assess whether the use of PRF facilitated graft uptake when compared to the traditional practice of using gel foam to augment graft uptake.

Methodology: A prospective and interventional randomized comparative study was conducted with 100 patients, who all underwent type 1 tympanoplasty using the temporalis fascia as the graft material through the underlay technique performed via a post-aural approach with PRF in one group (Group A) and without PRF in the other (Group B). Postoperative follow-up was done at the end of the first, second, and third months after surgery for the assessment of graft uptake, postoperative infection, and hearing status.

Results: The graft uptake rate in the two groups was 98% (Group A) and 88% (Group B) but was not statistically significant (p=0.11). The infection rate in the postoperative period was low in both groups, being 2% and 12% in Groups A and B, respectively. However, there was an improvement in the air-bone gap of 24.8 dB in the PRF group, while it was only 19 dB in the control group (p=0.008), which was statistically significant.

Conclusion: It was concluded from our study that PRF helps in better graft uptake and less postoperative infection rate and contributes to a marked enhancement in hearing.

## Introduction

Chronic otitis media (COM) is an inflammatory process involving the middle-ear cavity which results in permanent changes in the tympanic membrane, like atelectasis, dimer formation, perforation, tympanosclerosis, retraction pocket, or cholesteatoma. The global burden of COM is estimated to be around 65-300 million patients, out of which 60% experience significant hearing loss and contribute significantly to the burden of preventable hearing loss [1,2].

COM is pathologically divided into inactive and active mucosal and inactive and active squamous epithelial. Mucosal COM is thought to result from an incident of acute otitis media (AOM) in which the tympanic membrane ruptures with failure to repair or without any ongoing inflammation or an infection in the middle ear or mastoid. Such type of COM can remain inactive, can become active, or may occasionally even heal spontaneously [1]. The symptoms of mucosal COM include conductive hearing loss, painless, nonfoul-smelling aural discharge, aural fullness, and pulsatile tinnitus [3,4]. It can be managed medically and surgically. Medical management involves systemic and topical antibiotics with or without steroids. The surgical treatment for COM involves debridement of infective foci with restoration of hearing loss.

Tympanoplasty is a surgical procedure used to repair a perforated tympanic membrane, with or without ossicular reconstruction, to prevent reinfection and restore hearing ability using a graft material [5]. The temporalis fascia is the most effective and commonly used graft material due to its excellent properties, namely, anatomic proximity, translucency, and suppleness, which makes it the most commonly used graft material in various ear surgeries [6].

Increased experimental and clinical evidence identifies platelets as relevant modulators of various physio-pathologic processes, including inflammation and tissue regeneration. Various techniques of autologous platelet concentrates have been developed and are currently being used in oral and maxillofacial surgery worldwide due to their property of releasing growth factors playing a major role in wound healing. Platelet-rich fibrin (PRF), a second-generation platelet concentrate, is a unique platelet concentrate that was originally created in France by Choukroun et al. in 2001. It is produced by centrifuging the patient's blood without the use of any anticoagulant, overcoming the disadvantages and risks associated with using bovine thrombin in the preparation of platelet-rich plasma (PRP), a first-generation platelet concentrate [7].

PRF is composed of a close assembly of cytokines, glycan chains, structural glycoproteins, leukocytes, and biologically active proteins like platelet alpha granules, platelet-derived growth factors (PDGF), transforming growth factors (TGF), vascular endothelial growth factors (VEGF), and epidermal growth factors. They sequentially promote the revascularization and restoration of damaged connective tissues and proliferation and differentiation of mesenchymal stem cells and protect against infection [8]. Combined effects of growth factor secretion and fibroblast recruitment in PRF work synergistically to promote collagenogenesis and tissue regeneration, which makes it an ideal patch material for tympanic membrane perforation repair [9].

On the basis of available literature and its succinct review, we hypothesize that PRF is superior to gelatin sponge in type 1 tympanoplasty. Thus, through this study, we primarily aimed to assess the graft uptake with and without the use of PRF. Secondarily, we aimed to interrogate the rates of postoperative infection as well as the degree of improvement in hearing by assessing the changes in air-bone gap using audiometry. 

## Materials and methods

A prospective and interventional randomized comparative study was conducted in the Department of Otolaryngology-Head and Neck Surgery, Vardhman Mahavir Medical College and Safdarjung Hospital, New Delhi, India, over a period of 18 months from January 2021 to June 2022 after obtaining approval from the institute's Institutional Ethics Committee (approval number: IEC/VMMC/SJH/Thesis/2020-11/CC-89). A total of 100 patients with COM were divided into two groups with 50 participants each (Groups A and B). All patients underwent type 1 tympanoplasty using the temporalis fascia as the graft material through the underlay technique performed via a post-aural approach with PRF in Group A and without PRF in Group B. Postoperative follow-up was done at the end of the first, second, and third months after surgery for the assessment of graft uptake, postoperative infection, and hearing status.

Patients between 18 and 50 years of age as well as patients with unilateral dry, central tympanic membrane perforation were enrolled in the study. In contrast, patients with sensorineural hearing loss, active/inactive squamosal disease, ossicular deformity/marginal perforation, comorbidities like diabetes and hypertension, and a deranged coagulation profile were excluded from this study. Clinical Trials Registry-India (CTRI) registration was waived off for this study based on the expert opinion of the Institutional Ethics Committee.

Methodology

A detailed relevant history was taken, followed by a thorough general physical and otorhinolaryngological examination. The patients who fulfilled the study criteria were divided into two groups by an allocator who used a randomly selected block randomization technique to prevent any bias. The audiometric assessment was done using a pure tone audiometer to determine the extent of hearing loss preoperatively. This data was blinded to the surgeon performing the tympanoplasty. 

After taking proper informed written consent regarding the surgical procedure, patients were taken up for hematological investigations to ensure their eligibility for surgery. All patients (Group A and Group B) underwent type 1 tympanoplasty using the temporalis fascia as the graft material through the underlay technique with PRF in Group A and without PRF in Group B.

**Figure 1:**
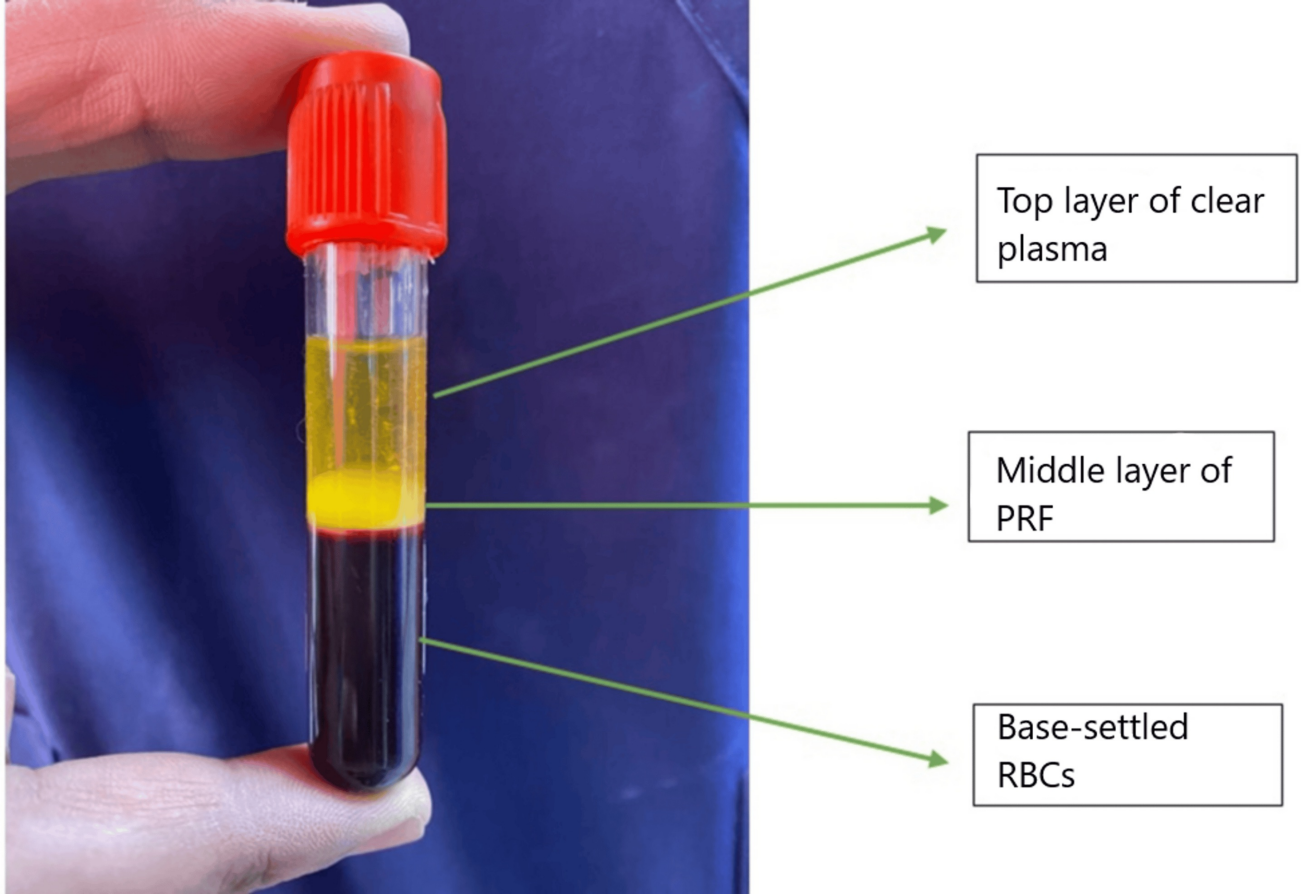
Test tube just after centrifugation, showing three layers: plasma, PRF, and RBCs PRF: platelet-rich fibrin; RBCs: red blood cells

**Figure 2:**
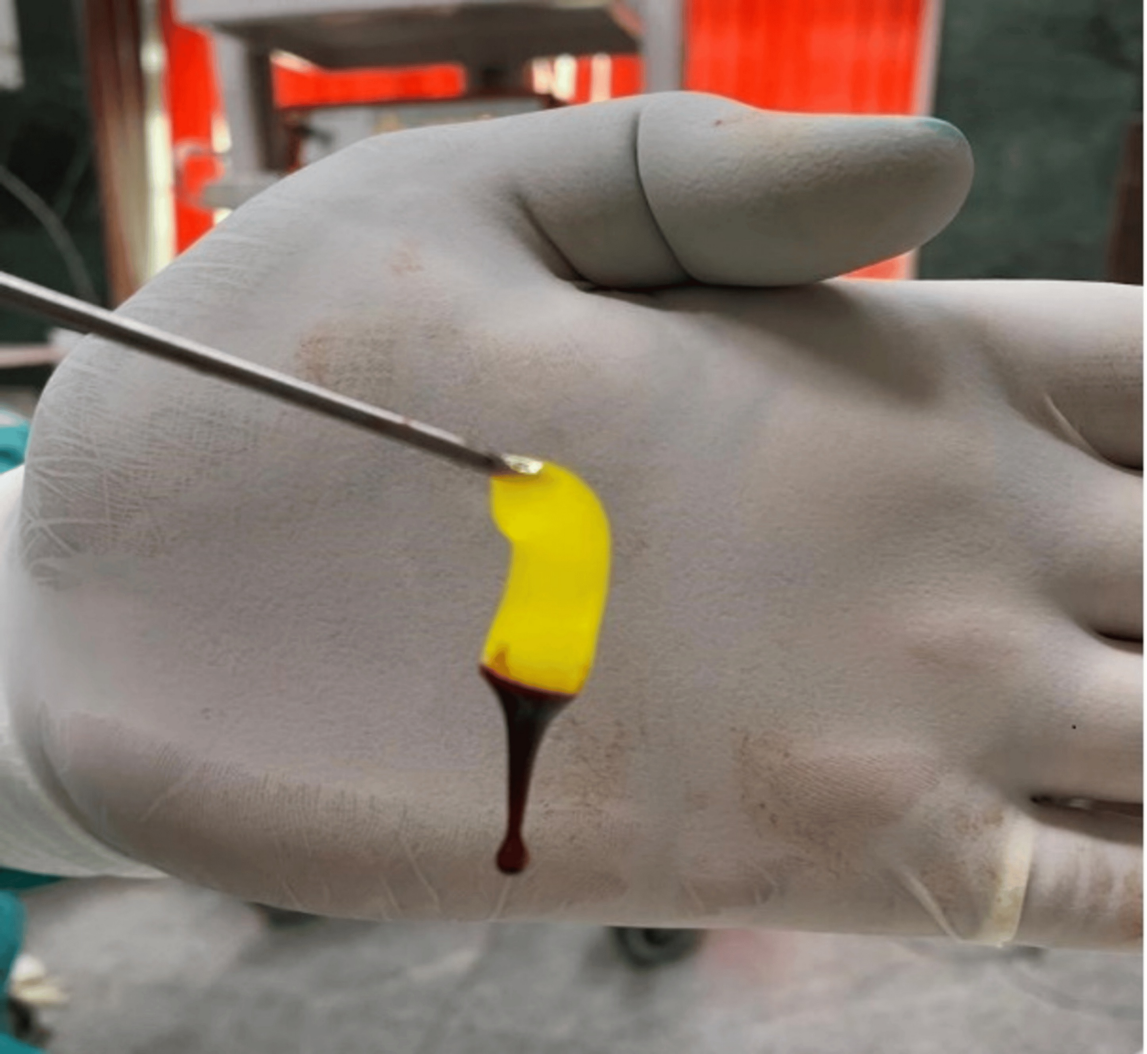
PRF plug held adjacent to the palm of the surgeon's hand PRF: platelet-rich fibrin

**Figure 3:**
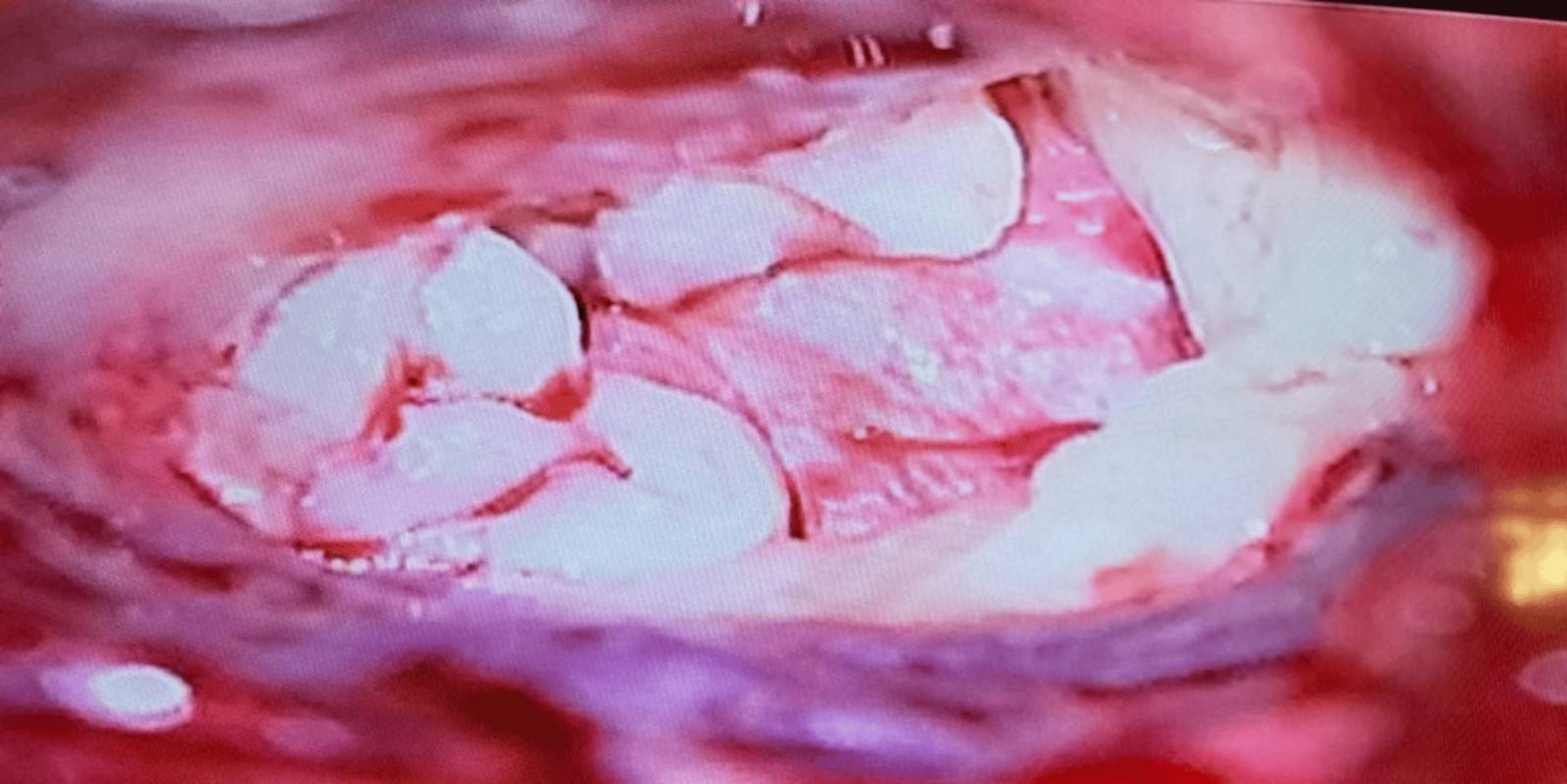
Intraoperative microscopic picture showing the PRF placed over the temporalis fascia graft PRF: platelet-rich fibrin

During the procedure, at the time of raising the flap, 10 ml of blood sample was withdrawn from the patient and collected in a plain vial. It was centrifuged immediately using a table-top centrifuge machine (REMI R-8C, Mumbai, India) at 2700 rpm for 10 minutes. After 10 minutes of centrifugation, the top layer of plasma was removed from the vial using a syringe and needle. A fibrin clot was obtained in the middle layer just above the red blood corpuscles settled at the bottom (Figure 1). This fibrin clot (PRF) was taken out using forceps under aseptic conditions (Figure 2). PRF was placed over the graft, after repositioning the tympanomeatal flap in Group A (Figure 3), and similarly, gel foam was used instead of PRF in Group B.

Both groups received oral antibiotics as per the routine protocol followed in our department. Patients were followed up at the first, second, and third months and were assessed by a blinded observer for graft uptake, postoperative infection, and improvement in hearing using a pure tone audiometer.

Otoendoscopic findings of the graft uptake, with the absence of any perforation, were taken as a successful surgical outcome, and failure of graft uptake or reperforation was taken as a poor surgical outcome. The presence of postoperative infection, as evidenced by purulent discharge in the canal, indicated poor surgical outcomes. Hearing assessment was done by comparing preoperative and postoperative pure tone audiometry (PTA) and tuning fork test (512 Hz).

Statistical analysis

The data entry and analysis were done using IBM SPSS Statistics for Windows, Version 25.0 (IBM Corp., Armonk, New York, United States). The categorical variables were assessed in the form of numbers and percentages, while quantitative data were presented in terms of mean±SD and median with 25th and 75th percentiles (interquartile range). The data normality was assessed using the Kolmogorov-Smirnov test. In cases where the data were not normal, non-parametric tests were used. Comparison of variables, which were quantitative and not normally distributed, was analyzed with the Mann-Whitney test. The Wilcoxon signed-rank test was used for comparison across interval follow-up periods. The nature of the qualitative variables was analyzed using the chi-squared test. Furthermore, if any cell had an expected value of <5, then Fisher's exact test was used. A p-value of <0.05 was considered statistically significant. 

## Results

A total of 100 patients aged 18-50 years diagnosed with COM were included in the study. After a complete otologic examination and preoperative PTA, patients underwent type 1 tympanoplasty via a post-aural approach using the temporalis fascia as the graft material. PRF was used as an adjunct in Group A, while Group B was subjected to gel foam. Patients were followed up at 30, 60, and 90 days postoperatively, and parameters such as postoperative infection rate, rate of graft uptake, and improvement in air-bone gap (dB) were marked among the two groups and subjected to statistical analysis using IBM SPSS Statistics for Windows, Version 25.0.

Demographic details

Of the total 100 patients enrolled in this study (Table 1), 57% were females (57/100), and 43% were males (43/100). Group A (underwent PRF) consisted of 30 females (60%) and 20 males (40%), while Group B (underwent gel foam therapy) consisted of 27 (54%) females and 23 (46%) males. The age range among the two groups was similar and ranged from 19 to 50 years in Group A and 18 to 48 years in Group B. 

**Table 1:** Demographics regarding patient age distribution and gender distribution among the two groups

	Group A (n=50)	Group B (n=50)	Total
Age (years) with mean±SD distribution and gender (female/male) distribution			
18-20	6 (12%)	5 (10%)	11 (11%)
21-30	17 (34%)	15 (30%)	32 (32%)
31-40	15 (30%)	17 (34%)	32 (32%)
41-50	12 (24%)	13 (26%)	25 (25%)
Mean±SD	33.04±9.1	33.42±8.6	3.23±8.8
Median (25th-75th percentiles)	34 (26-39.75)	34.5 (2.25-40.75)	34 (26-40.2)
Range	19-50	18-48	18-50
Gender			
Female	30 (60%)	27 (54%)	57 (57%)
Male	20 (40%)	23 (46%)	43 (43%)

Comparison of postoperative infection rates

While there was no statistical significance between the two groups (Table 2) in terms of infection rates at the 30-day post-op follow-up, there were no cases of infection in Group A that was subjected to PRF, while 4/50 (8%) of Group B patients had an episode of culture-positive otitis media. The distribution of postoperative infection at 90 days was less in Group A but not statistically significant (p=0.112). 

**Table 2:** Comparison of postoperative infection rates between the two groups at the 30-, 60-, and 90-day post-op follow-ups Fisher's exact test was used to assess the significant association between two groups among the variables of 30, 60, and 90 days postoperative infection.

	Group A (n=50)	Group B (n=50)	Total	P-value
Postoperative infection at 30 days				
No	50 (100%)	46 (92%)	96 (96%)	0.117
Yes	0 (0%)	4 (8%)	4 (4%)	
Total	50 (100%)	50 (100%)	100 (100%)	
Postoperative infection at 60 and 90 days				
No	49 (98%)	44 (88%)	93 (93%)	0.112
Yes	1 (2%)	6 (12%)	7 (7%)	
Total	50 (100%)	50 (100%)	100 (100%)	

Comparison of graft uptake at 30, 60, and 90 days post-op between Groups A and B

Table 3 depicts that 1/50 (2%) patient from Group A did not report graft uptake at 30, 60, or 90 days, indicating that the graft is best suited to uptake within the first 30 days, following which its uptake remains questionable. The remaining 49/50 (98%) patients objectively showed graft uptake on all three follow-ups. Similarly, in Group B, 6/50 (12%) patients did not show any graft uptake and remained void of any changes on the subsequent follow-ups at 60 and 90 days.

**Table 3:** Comparison of graft uptake among the two groups at 30, 60, and 90 days Fisher's exact test was used to derive the statistical significance between the two groups.

Graft uptake at 30, 60, and 90 days	Group A (n=50)	Group B (n=50)	Total	P-value
No	1(2%)	6 (12%)	7 (7%)	0.112
Yes	49 (98%)	44 (88%)	93 (93%)	
Total	50 (100%)	50 (100%)	100 (100%)	

Distribution of graft uptake at 30, 60, and 90 days was better in Group A but not statistically significant (p=0.112).

Comparison of air-bone gap between the two groups

While there was no statistically significant difference between the two groups in terms of reduction in air-bone gap post-tympanoplasty, within the groups (Table 4), there was significant improvement (p<0.0001) with Group A patients having their mean air-bone deficit reduced from a mean±SD of 45.5±12.1 dB preoperatively to a mean±SD of 20.7±9.4 dB, indicating a significant improvement postoperatively. Similarly, in Group B, the preoperative mean±SD air-bone gap reduced from 43.1±12.5 to 24.1±13.8 dB, indicating a significant improvement postoperatively (intragroup p<0.0001).

**Table 4:** Comparison of air-bone gap (dB) between the two groups The Mann-Whitney test and Wilcoxon signed-rank test were used to assess the significance of the association between variables.

Air-bone gap (dB)	Group A (n=50)	Group B (n=50)	Total	P-value
Preoperative				
Mean±SD	45.5±12.1	43.1±12.57	44.3±12.3	0.331
Median (25th-75th percentiles)	45 (35-55)	45 (30-50)	45 (30-50)	
Range	25-70	25-70	25-70	
Postoperative				
Mean±SD	20.7±9.4	24.1±13.8	22.4±11.8	0.479
Median (25th-75th percentiles)	20 (15-28)	20 (15-30)	20 (15-30)	
Range	10-50	10-60	10-60	
Intragroup p-value	<0.0001	<0.0001	-	-

## Discussion

A significant number of graft failures postoperatively, along with a significant postoperative infection rate, paved the way for the creation of numerous procedures or biomaterials for use in tympanoplasty in order to boost graft uptake and lower postoperative infection rates. Choukroun et al. created PRF, a second-generation platelet derivative that featured numerous growth factors and was high in leukocytes, as part of the development of new methods and biomaterials to speed healing [7].

Epidemiology

In our study, the age distribution was comparable between Groups A and B (Table 1). The median age (years) in Group A was 34 years, and in Group B, it was 34.5 years, with no significant difference between them. This was concordant with the results published by Nair et al., wherein the average age was 39.91 years, while it was 29.12 years in the control group [2].

The study showed that there were 60% females and 40% males in Group A and 54% females and 46% males in Group B (Table 1), showing a female preponderance as in previous studies, which may be considered an indicator of increased treatment-seeking behavior among young and female patients [2,10].

Infection

In our study, 98% of patients in Group A had no postoperative infection, whereas in Group B, 88% of patients had no postoperative infection at the end of the three-month postoperative period (Table 2). While Nair et al. concluded that the rate of postoperative infection in myringoplasty using PRF was 4.7% and without PRF was 19% [2], in our study, though not statistically significant, we found a decrease in postoperative infection rate with the use of PRF. Multiple studies [11,12] showed a statistically significant decrease in postoperative infection rates. Thus, the use of PRF in tympanoplasty reduces the rate of postoperative infection due to its biologically active proteins and growth factors and high concentration of white blood cells [8].

Graft uptake

The postoperative successful graft uptake in our study after one, two, and three months (Table 3) in cases was 98%, while in the control group, it was 88%, which is comparable to previous studies [13-15]. One study showed that 97.7% of patients had successful graft uptake with PRF, while only 81% of controls had successful graft uptake where PRF helped in improved perforation closure [2]. The results of our study are concordant with earlier results published by Gökçe Kütük and Özdaş who showed successful graft uptake in 94.4% of the cases and 74.5% of the controls [16]. A study by Hosam et al. showed graft uptake in 96% of the patients in the study group compared to 76% in the control group [15]. Another study showed that the success rate of graft uptake in tympanoplasty with PRF was 96%. It also revealed that PRF cannot reverse an unsatisfactory surgical approach; however, it does provide mechanical and inflammatory protection for the tympanic graft and hastens matrix remodeling and cell proliferation [14].

Hearing improvement

In our study, there was a significant decrease in air-bone gap (dB) in the postoperative period as compared to the preoperative period in both groups (p<0.0001) (Table 4). The median of improvement in air-bone gap (dB) in Group A was 25 (15-30) which was significantly higher than that of Group B with a p-value of 0.008 suggesting more improvement in hearing in Group A in comparison to Group B. In a study done by El-Anwar et al., the mean air-bone gap at speech frequencies improved significantly from 16±3.83 dB preoperatively to 7±2.9 dB postoperatively (t=9.3671; p<0.001), thus concluding that PRP myringoplasty is a safe and effective procedure that is suitable for repairing small perforations [12]. A study of trans-canal type 1 tympanoplasty with and without PRF conducted on 82 patients demonstrated a hearing improvement of 78% in the PRF group as compared to 46.3% in the non-PRF group. Thus, in comparison with the previous studies [13,17], our study also concluded that PRF contributes to a marked enhancement in hearing following tympanoplasty.

Using PRF in type 1 tympanoplasty with the temporalis fascia as the graft material helps augment healing and shows better graft uptake rates along with a decrease in postoperative infection rates. It also showed a significant improvement in hearing in the group in which PRF was used in comparison to the other group. Further studies with a larger sample size are required to study graft uptake in patients using PRF. Since PRF is autologously prepared and more beneficial, it can thus be used as a standard operating procedure with better surgical outcomes.

Limitations

The first and foremost limitation of our study was the small sample size as well as the short duration of follow-ups. Furthermore, while the results of our study show no statistically significant correlation between graft uptake rates and postoperative infection rates, there were, however, reduced postoperative infection rates at the 30- and 90-day postoperative period in the group where PRF was used, suggesting interval improvement in post-op infection incidence. While this might not be completely attributable to the effects of PRF, we advise large-scale multicenter trials with longer follow-up periods to validate the results of our study and their incorporation into routine clinical practice.

## Conclusions

Our study was conducted to assess the effect of PRF in type 1 tympanoplasty to aid graft uptake. One hundred patients were enrolled in the study and divided into two groups, with Group A patients subjected to PRF-aided type 1 tympanoplasty and Group B patients to plain gel foam. Patients were reviewed at 30, 60, and 90 days, and it was seen that graft uptake rates at the end of the first, second, and third months in Groups A and B were 98% vs. 88%, respectively, showing a significant increase in the graft uptake rates using PRF. While these results were not statistically significant, they imply a preferential inclusion of PRF as an adjunct to aid graft uptake in type 1 tympanoplasty. 

We also assessed the postoperative infection rates between the two groups and demonstrated that the distribution of postoperative infection at each follow-up was comparable between Groups A and B at 0% vs. 8%, respectively (p=0.117), suggesting no significant correlation between reduced postoperative infection rates using either technique, thereby proving that neither technique is superior to the other.

These results encourage further large-scale multicenter studies to establish the validity of our findings, and while our results were not statistically significant, they indicate that the role of PRF in aiding graft uptake, as established in the literature, is not as straightforward as thought and warrants a detailed workup with long-term follow-ups to establish its efficacy. 
